# Ammonium tetrathiomolybdate triggers autophagy-dependent NRF2 activation in vascular endothelial cells

**DOI:** 10.1038/s41419-022-05183-z

**Published:** 2022-08-25

**Authors:** Mengling Zhang, Hongmei Qiu, Lejiao Mao, Bin Wang, Na Li, Yinzhen Fan, Ping Weng, Siyao Hu, Xiaomei Dong, Xia Qin, Chengzhi Chen, Zhen Zou, Chao Yu, Jun Zhang

**Affiliations:** 1 Chongqing Key Laboratory for Pharmaceutical Metabolism Research, College of Pharmacy, Chongqing Medical University, 400016 Chongqing, People’s Republic of China; 2 Molecular Biology Laboratory of Respiratory Disease, Institute of Life Sciences, Chongqing Medical University, 400016 Chongqing, People’s Republic of China; 3 Department of Pharmacy, The First Affiliated Hospital of Chongqing Medical University, 400016 Chongqing, People’s Republic of China; 4 Department of Occupational and Environmental Health, School of Public Health, Chongqing Medical University, 400016 Chongqing, People’s Republic of China; 5 Research Center for Environment and Human Health, School of Public Health, Chongqing Medical University, 400016 Chongqing, People’s Republic of China

**Keywords:** Drug development, Cell death

## Abstract

Ammonium tetrathiomolybdate (TTM) is a copper chelator in clinical trials for treatment of Wilson’s disease, tumors and other diseases. In the current study, we innovatively discovered that TTM is a novel NRF2 activator and illustrated that autophagy contributed to TTM-induced NRF2 activation. We showed that TTM treatment promoted NRF2 nuclear translocation and upregulated transcription level of NRF2 target genes including *HMOX1*, *GCLM*, and *SLC7A11* in vascular endothelial cells (HUVECs). Moreover, NRF2 deficiency directly hindered TTM-mediated antioxidative effects. Followingly, we revealed that overexpression of KEAP1, a negative regulator of NRF2, significantly repressed NRF2 activation induced by TTM. Further mutation analysis revealed that KEAP1 Cys151 is a major sensor responsible for TTM-initiated NRF2 signaling, suggesting that KEAP1 is involved in TTM-mediated NRF2 activation. Notably, we found that TTM can trigger autophagy as evidenced by accumulation of autophagosomes, elevation of LC3BI-II/I, increase of LC3 puncta and activation of AMPK/mTOR/ULK1 pathway. Autophagic flux assay indicated that TTM significantly enhanced autophagic flux in HUVECs. Inhibition of autophagy with knockout of autophagy key gene *ATG5* resulted in suppression of TTM-induced NRF2 activation. TTM also induced phosphorylation of autophagy receptor SQSTM1 at Ser349, while SQSTM1-deficiency inhibited KEAP1 degradation and blocked NRF2 signaling pathway, suggesting that TTM-induced NRF2 activation is autophagy dependent. As the novel NRF2 activator, TTM protected against sodium arsenite (NaAsO_2_)-induced oxidative stress and cell death, while NRF2 deficiency weakened TTM antioxidative effects. Finally, we showed that autophagy-dependent NRF2 activation contributed to the protective effects of TTM against NaAsO_2_-induced oxidative injury, because of *ATG5* or *SQSTM1* knockout aggravated NaAsO_2_-induced elevation of HMOX1, cleaved PARP and γH2AX. Taken together, our findings highlight copper chelator TTM is a novel autophagy-dependent NRF2 activator and shed a new light on the cure for oxidative damage-related diseases.

## Introduction

Ammonium tetrathiomolybdate (TTM) is a copper chelator in clinical trials for treatment of Wilson’s disease, an autosomal recessive inherited disorder of copper metabolism [1]. Surprisingly, growing evidences have shown that TTM has many other potential clinical applications. For instance, TTM can inhibit tumor growth and angiogenesis and is regarded as a potential drug for tumor clinical therapy [2–5]. It has also been demonstrated that TTM can be used as an effective therapeutic agent against Alzheimer’s disease through promoting non-amyloidogenic processing of amyloid-β precursor protein [6]. Given its excellent anti-inflammatory properties, TTM can mitigate cardiovascular risk factors-induced endothelial dysfunctions and then prevent the development of cardiovascular disease including atherosclerosis [7], pulmonary arterial hypertension [8] and abdominal aortic aneurysm [9]. Nevertheless, the molecular mechanism underlying the protective effects of TTM has not yet been fully elucidated.

Reactive oxygen species (ROS) are a group of active molecules including superoxide anions, hydrogen peroxide, hydroxyl radical and other highly reactive molecules, which derived from molecular oxygen during reduction-oxidation (redox) reactions. Intracellular ROS homeostasis is crucial for cell metabolism and cell fate. Low levels of ROS are essential for triggering cell protective machineries including antioxidant and anti-inflammatory activities. However, excessive ROS result in increased oxidative stress in cell and induce oxidation of cellular macromolecules such as nucleic acids, proteins and lipids [10]. Extensive studies have revealed that oxidative stress and vascular inflammation are two major molecular mechanisms contributing to vascular injury and increased cardiovascular disease risk [11].

Nuclear factor erythroid 2-related factor 2 (NFE2L2, also named as NRF2) is a master antioxidant response regulator that transcriptionally regulates many antioxidant proteins including heme oxygenase 1 (HMOX1), glutamate-cysteine ligase modifier subunit (GCLM), sequestosome 1 (SQSTM1) and solute carrier family 7 member 11 (SLC7A11). Emerging studies has demonstrated that NRF2 maintains cellular redox homeostasis in cardiovascular system and protects against endothelial dysfunction and vascular disease. Aging-induced NRF2 dysfunctions disrupted cellular oxidative and inflammatory balance and consequently caused vascular cognitive impairment and dementia [12]. In addition, NRF2 deficiency obviously induced atherosclerotic plaque instability through triggering systemic inflammation and oxidative stress in hypercholesterolemic mice [13]. Conversely, Dai et al. demonstrated that biomechanical forces activated NRF2 signaling via phosphoinositol 3-kinase/AKT-dependent pathway, which regulated endothelial cells redox homeostasis and attenuated high fat diet-induced atherosclerosis [14]. These data indicate that NRF2 is a potential therapeutic target for treatment of oxidative stress-induced vascular disease.

Currently, multiple pharmacologic inducers of NRF2 are subjected to clinical trials, which shed a new light on treatment for cardiovascular diseases and other diseases [15, 16]. For example, sulforaphane is an electrophilic NRF2 activator that plays a protective role against COPD, angiotensin II-induced cardiomyopathy and other diseases [17–19]. Resveratrol, a polyphenolic NRF2 inducer derived from grapes, improves vascular functions in hypertensive patients and inhibits atherosclerosis by reducing expression of intercellular adhesion molecule-1 [20, 21]. Recently, Ryo Kurosawa et al. screened thousands of compounds from original library and identified celastramycin as a novel NRF2 activator, which could obviously reduce cellular ROS levels and ameliorate pulmonary arterial hypertension [22]. Despite this, it is meaningful to find safer and more effective NRF2 inducer that have entered clinical evaluation for treatment of cardiovascular disease.

In this work, we demonstrate that clinical copper chelator TTM is a novel NRF2 activator which increases NRF2 protein level and transcriptionally activates NRF2 downstream antioxidant molecules. Mechanistically, TTM enhances autophagic flux via AMPK/mTOR/ULK1 pathway, and then promotes degradation of negative regulator KEAP1 by autophagy. In addition, we show that TTM inhibits oxidative stressor NaAsO_2_-induced oxidative injury and cell death in HUVECs. These findings highlight copper chelator TTM is a novel NRF2 activator and shed a new light on oxidative damage and cardiovascular disease.

## Materials and methods

### Regents

Ammonium tetrathiomolybdate (TTM, #323446) and chloroquine diphosphate salt (CQ, #C6628) were purchased from Sigma-Aldrich (St. Louis, MO, USA). MG132 (#S1748) and dihydroethidium (DHE, #S0063) were obtained from Beyotime (Shanghai, China). Tert-butylhydroquinone (tBHQ, #HY-100489) was purchased from MedChemExpress (Shanghai, China). Bafilomycin A1 (BafA1, #sc-201550) was purchased from Santa Cruz Biotechnology (Santa Cruz, CA, USA). Sodium arsenite (NaAsO_2_, #H4525) was obtained from Xiya Reagent (Shandong, China). 7-aminoactinomycin D (7-AAD, #AP104) and Annexin V FITC/ Propidium iodide (PI) apoptosis kit (#70-APCC101-100) were obtained from MultiSciences (Hangzhou, China). Dulbecco’s Modified Eagle Medium (DMEM, #C11995500BT) was purchased from Gibco (Grand Island, NY, USA). Fetal bovine serum (FBS, #S711-001S) was purchased from Lonsera (Shanghai, China). Penicillin-streptomycin (#15140122) was obtained from Thermo Fisher Scientific (Waltham, MA, USA). Puromycin (#P8230) was purchased from Solarbio (Beijing, China). CellTiter 96® Aqueous One Solution Cell Proliferation Assay (MTS) kit (#G3581) was obtained from Promega (Madison, WI, USA).

### Cell culture

Human umbilical vein endothelial cell line (HUVECs, CRL-1730), a well-accepted vascular endothelial cell model, was obtained from American Type Culture Collection (Manassas, VA, USA). Human embryonic kidney 293T/17 (HEK293T/17) cells and human cervical cancer cell line Hela were purchased from National Collection of Authenticated Cell Cultures (Shanghai, China). All cells were cultured in Dulbecco’s modified Eagle’s medium (DMEM) supplemented with 10% (vol/vol) FBS and 100 U/mL penicillin-streptomycin in 5% CO_2_ incubator at 37 °C. All cell lines are negative for mycoplasma contamination and confirmed by Short Tandem Repeat (STR) DNA profiling.

### Stable cell line construction

The HUVECs RFP-GFP-LC3 reporter cell line was established as described in our previous study [23]. Gene knockout cell lines are constructed using CRISPR/Cas9 system. The CRISPR/Cas9 lentivirus plasmid lentiCRISPRv2 (Plasmid #52961) was purchased from Addgene (Watertown, MA, USA). The small guide RNAs (sgRNAs) targeting *NRF2*, *ATG5* and *SQSTM1* were synthesized and inserted into *BsmB*I-digested lentiCRISPRv2. The lentivirus plasmids were co-transfected with lentivirus packaging plasmids psPAX2 (Plasmid #12260, Addgene) and pMD2.G (Plasmid #12259, Addgene) with 4:3:2 ratio using Neofect™ DNA transfection reagent (Neofect, Beijing, China). Then, HUVECs were infected with filtered lentivirus supernatant and selected with puromycin (10 μg/ml). The knockout efficiency was validated by Sanger sequencing and western blotting.

### MTS cell viability assay

Cells were seeded in wells of a 96-well tissue culture plate for overnight and then treated with thiol-reactive oxidative stressor NaAsO_2_ with or without copper chelator TTM for 24 h. Cell viability was detected using CellTiter96® Aqueous One Solution Cell Proliferation Assay Kit (#G3582, Promega) in a microplate reader (Molecular Devices Corp, Sunnyvale, CA, USA) according to the manufacture’s protocol.

### Animal treatment and immunohistochemistry

Female C57BL/6J mice (age 6–8 weeks, weight 18–20 g) were obtained from Experimental Animal Center of Chongqing Medical University. Animal experiments were approved by the Institutional Animal Care and Use Committee of Chongqing Medical University. The mice were randomly divided into two groups (8 mice per group): Control group and TTM group. After a week adaption, the TTM treatment group of mice were gavaged with 30 mg/kg of TTM twice a day for 7 consecutive days. The mice were killed and the liver were obtained for further immunohistochemistry assay as described in our previous study [24].

### Western blotting

Cells were washed with phosphate-buffered saline (PBS) and lysed with 2×sodium dodecyl sulfate (SDS) loading buffer (0.5% sucrose, 0.2% bromophenol blue, 5% β-mercaptoethanol and 2% SDS) for 15 min at 4 °C. The liver tissues were homogenized by a tissue homogenizer in cold RIPA lysis buffer (Cat# P0013B, Beyotime) and centrifuged at 14,000×*g* for 15 min at 4 °C. The supernatants were collected for western blotting. All lysates were heated in a metal bath for 5 min at 100 °C and subjected to western blotting. The following primary antibodies were used: NRF2 (#16396-1-AP, 1:1,000, Proteintech), KEAP1 (#8047S, 1:3,000, Cell Signaling Technology), HMOX1 (#66743-1-Ig, 1:3,000, Proteintech), GCLM (#A5939, 1:3,000, Bimake), SLC7A11 (#ab175186, 1:1,000, Abcam) LC3B (#L7543, 1:3,000, Sigma), SQSTM1 (#18420-1-AP, 1:3,000, Proteintech), p-SQSTM1 (Ser349) (#16177, 1:1000, Cell Signaling Technology), ATG5 (#9980S, 1:3,000, Cell Signaling Technology), p-mTOR (Ser2448) (#5536S, 1:1000, Cell Signaling Technology), p-AMPKα (Thr172) (#2535T, 1:1,000, Cell Signaling Technology), p-ULK1 (Ser757) (#14202T, 1:1,000, Cell Signaling Technology), Cleaved PARP (#5625, 1:1,000, Cell Signaling Technology), γH2AX (#9718, 1:3,000, Cell Signaling Technology), GAPDH (#60004-1-Ig, 1:6,000, Proteintech) and β-Actin (#HC201-01, 1:10,000, TransGen). The following secondary antibodies were used: HRP-conjugated goat anti-rabbit IgG (#7074S, 1:10,000, Cell Signaling Technology) and HRP-conjugated goat anti-mouse IgG (#7076S, 1:10,000, Cell Signaling Technology). The Image J software (NIH, Bethesda, MD, USA) was used for quantification of band intensity of western blotting. All full and uncropped western blotting bands are uploaded as “Supplementary Material-Original Western Blotting Bands”.

### Quantitative PCR Assay

Total RNA was extracted using Eastep®Super Total RNA Extraction Kit (#LS1040, Promega) and then reversely transcribed into cDNA using Hiscirpt® II Q RT SuperMix for quantitative PCR (qPCR; +gDNA wiper) Kit (#R233-01, Vazyme, Nanjing, China) according to the manufacturer’s instructions. qPCR was performed using ChamQ Universal SYBR qPCR Master Mix (#Q711-02/03, Vazyme) under a CFX96 Touch Real-Time PCR Detection System (Bio-Rad, Hercules, CA, USA). The results were calculated using the 2^-ΔΔCt^ method.

### Immunofluorescence assay

Cells were seeded on glass coverslips in 24-well plates for overnight and then treated with 100 μM TTM for 12 h. Followingly, cells were fixed with cold 4% paraformaldehyde for 15 min and permeabilized with 0.2% Triton X-100 for 15 min at room temperature. After washing with PBS, cells were blocked with 2% bovine serum albumin for 1 h at room temperature and then incubated with primary antibody against NRF2 (#16396-1-AP, 1:100, Proteintech) at 4 °C for overnight. After washing three times with PBS, the cells were incubated with Alexa Fluor 594-conjugated donkey anti-rabbit IgG secondary antibodies (#A-21207, 1:500, Thermo Fisher Scientific) and 4′,6-Diamidino-2-Phenylindole, Dilactate (DAPI, #D3571, Invitrogen) for 1 h at room temperature. Finally, coverslips were rinsed with PBS and sealed with nail polish. The coverslips were observed under a Nikon A1R confocal microscope (Nikon, Tokyo, Japan).

### Transmission electron microscope

After treatment with TTM, cells were detached by trypsin/EDTA and centrifuged for 5 min at 100×g. Next, the cell pellets were incubated with 4% glutaraldehyde followed by 1% osmium tetroxide. After dehydration in a graded series of alcohol and acetone, cells were embedded in Epon 812 (Electron Microscopy Sciences, Hatfield, PA, USA). Ultrathin sections were cut on a Leica EM UC7 Ultramicrotome (Leica, Wetzlar, Germany), and then poststained with uranyl acetate and lead citrate. Transmission electron microscope (TEM) images were taken under a JEM-1400 Plus transmission electron microscope (JEOL Ltd. Tokyo, Japan).

### Small interfering RNA transfection

HUVECs were seeded in a 12-well plate for overnight and then transfected negative control siRNA (siGFP, GCAGCACGACUUCUUCAAGUU) or siRNA targeting *NRF2* (si*NRF2*, GGUUGAGACUACCAUGGUU) for 48 h using RNAi-Mate (#G04001, Gene Pharma, Shanghai) according to the manufacturer’s instruction. RNAi efficiency was detected with western blotting analysis.

### Fluorescence activated cell sorting

After treatment, cells were digested by trypsin/EDTA and collected by centrifugation for 5 min at 100 × *g*. Then, cells were stained with DHE, 7-AAD and Annexin V-FITC/PI fluorescent probes for 15 min at 4 °C in the dark, respectively. The stained cells were detected under a CytoFLEX flow cytometry (Beckman Coulter, Miami, FL, USA). All Fluorescence activated cell sorting (FACS) data were analyzed by FlowJo™ v10 Software BD Biosciences (San Jose, CA, USA).

### Statistical analysis

Each experiment was repeated for at least three times with similar results obtained. Data are presented as mean ± standard deviation (S.D.). Unpaired Student’s *t* test and one-way ANOVA followed by Tukey multiple comparison test were used for statistical analysis in this study. *p < 0.05 was considered statistical significance. All statistical tests were performed using GraphPad Prism 9.3 (GraphPad Software, San Diego, CA, USA).

## Results

### Copper chelator TTM activates NRF2 antioxidative signaling in vascular endothelial cells

**Fig. 1:**
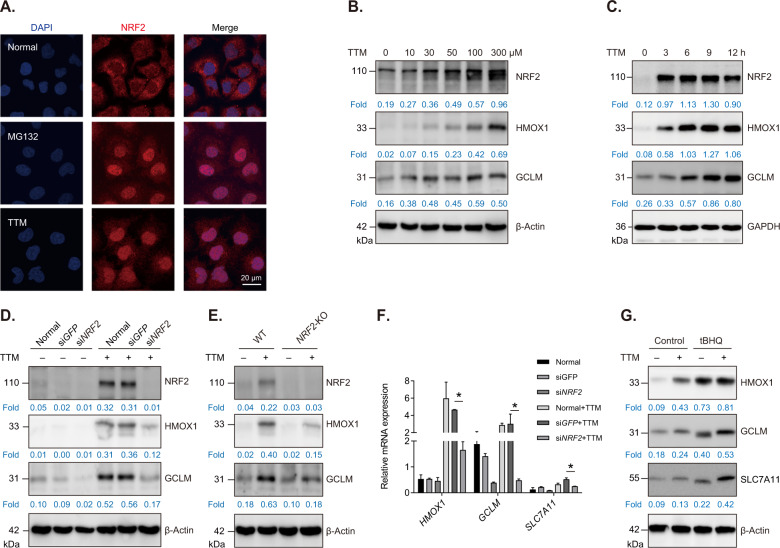
Copper chelator TTM activates NRF2 antioxidative signaling in HUVECs. **A** Immunofluorescence analysis of NRF2 in HUVECs after treatment with 100 μM TTM for 12 h. MG132 was used as a positive control. The nucleus was stained with DAPI. Scale bar, 20 μm. **B** Western blot analysis and quantification of NRF2, HMOX1 and GCLM in HUVECs treated with different dose of TTM for 12 h. β-Actin was used as loading control. **C** Western blot analysis and quantification of NRF2, HMOX1, GCLM and GAPDH in HUVECs treated with 100 μM TTM for indicated time. **D** Western blot analysis and quantification of NRF2, HMOX1, GCLM and β-Actin (loading control). HUVECs were transfected with si*NRF2* or si*GFP* (control) for 48 h and then treated with 100 μM TTM for 12 h. **E** Western blot analysis and quantification of NRF2, HMOX1, GCLM and β-Actin in TTM-treated normal (WT) or *NRF2* knockout (*NRF2* KO) cells. **F** Quantification of mRNA levels of *HMOX1*, *GCLM* and *SLC7A11* in si*NRF2*-transfected cells. One-way ANOVA followed by a Tukey multiple comparison test was used for statistical analysis. *p < 0.05. **G** Western blot analysis and quantification NRF2 downstream proteins including HMOX1, GCLM and SLC7A11 in TTM-treated HUVECs with or without 10 µM tBHQ for 12 h.

Previous studies have revealed that tetrathiomolybdate (TTM), a specific copper chelator, is able to alleviate vascular injury and inhibit atherosclerotic lesion development in apolipoprotein E-deficient mice [7, 25]. In this study, we firstly found that TTM activated NRF2 antioxidative signaling as evidenced by an increase of nuclear localization of NRF2 in TTM-treated HUVECs (a human endothelial cell line; Fig. 1A). We also showed that TTM increased NRF2 levels and upregulated its downstream antioxidant proteins including HMOX1 and GCLM in HUVECs (Fig. 1B, C). Moreover, we investigated the effects of TTM on NRF2 activation in mice model and showed that TTM treatment obviously increased protein levels and nuclear translocation of NRF2 in liver blood vessels (Fig. S1B, C). To further verify TTM regulates NRF2 signaling, *NRF2* mRNA levels were knocked down using small interfering RNA (siRNA). Results showed that the upregulation of HMOX1 and GCLM induced by TTM was obviously inhibited in si*NRF2*-transfected cells (Fig. 1D). Moreover, we showed that *NRF2* knockdown suppressed transcription of NRF2 target genes including *HMOX1*, *GCLM*, and *SLC7A11*, suggesting TTM treatment indeed triggers NRF2 signaling pathway in HUVECs (Fig. 1F). In addition, we constructed a *NRF2* knockout cell line (*NRF2*-KO) using CRISPR/Cas9 and confirmed that *NRF2* knockout remarkably decreased protein levels of HMOX1 and GCLM induced by TTM (Fig. 1E). Finally, we further revealed that protein levels of NRF2 downstream molecules were intensely upregulated in tBHQ (an activator of NRF2)-treated HUVECs (Fig. 1G). These data suggest copper chelator TTM is a novel NRF2 activator and has potential in treatment for oxidative stress-induced cardiovascular diseases.

### KEAP1 is implicated in NRF2 activation in TTM-treated vascular endothelial cells

**Fig. 2:**
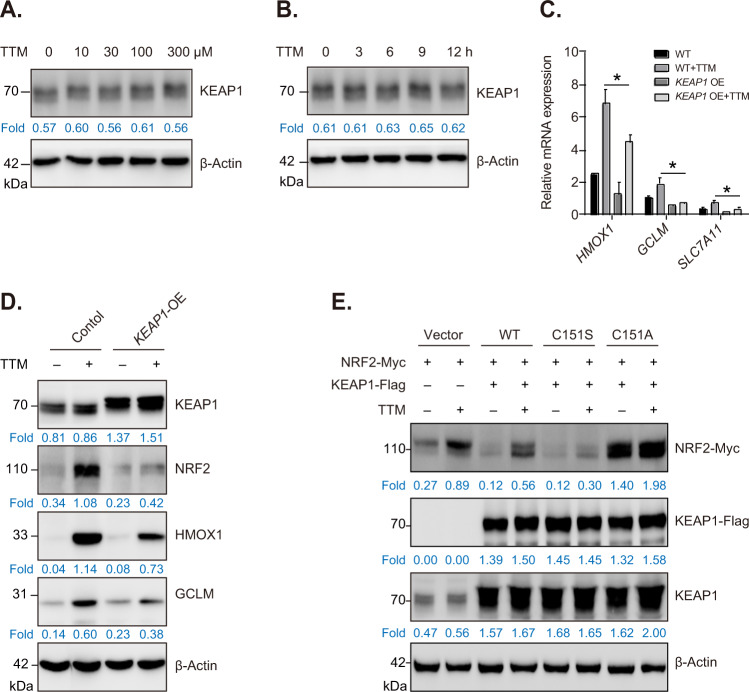
KEAP1 is involved in NRF2 pathway activation in TTM-treated HUVECs. **A** Western blot analysis and quantification of KEAP1 in 0, 10, 30, 100, or 300 μM TTM-treated HUVECs. β-Actin was used as loading control. **B** Western blot analysis and quantification of KEAP1 and GAPDH in HUVECs treated with TTM (100 μM) for 0, 3, 6, 9, and 12 h, respectively. **C** qPCR analysis mRNA levels of *HMOX1*, *GCLM*, *SLC7A11* in *KEAP1*-OE cells treated with or without TTM. One-way ANOVA followed by a Tukey multiple comparison test was used for statistical analysis. *p < 0.05. **D** Western blot analysis and quantification of KEAP1, NRF2, HMOX1, and GCLM in *KEAP1*-OE cells treated with or without TTM. β-Actin was used as loading control. **E** Plasmid vectors expressing KEAP1-Flag (WT), KEAP1-Flag (C151S) or KEAP1-Flag (C151A) were co-transfected with NRF2-Myc into Hela cells for 48 h and then treated with or without 100 μM TTM for 12 h. Western blot analysis and quantification of protein levels of exogenous NRF2-Myc, KEAP1-Flag and endogenous KEAP1. β-Actin was used as loading control.

It is well documented that KEAP1 acts as a negative regulator of NRF2 signaling. To confirm whether KEAP1 is implicated in TTM-regulated NRF2 activation, we firstly detected protein levels of KEAP1 in TTM-treated HUVECs. Our results showed that TTM treatment did not affect protein levels of KEAP1 in HUVECs (Fig. 2A, B). Despite this, we found that KEAP1 overexpression (*KEAP1* OE) considerably prevented transcriptional upregulation of NRF2 target gene including *HMOX1*, *GCLM* and *SLC7A11* induced by TTM (Fig. 2C). Meanwhile, *KEAP1* OE prevented the activation of NRF2 signaling and decreased protein levels of HMOX1 and GCLM in TTM-treated cells (Fig. 2D). Keap1 cysteine 151 (C151) is crucial for KEAP1-NRF2 interaction [26]. The substitution of C151 to serine (C151S) reduces KEAP1 sensitivity to oxidative or electrophilic stimuli, consequently stabilizing KEAP1-NRF2 complex and leading to degradation of NRF2 in ubiquitin-proteasome system. On the contrary, the substitution of C151 to alanine (C151A) facilitates NRF2 release from KEAP1-NRF2 complex and promotes NRF2 signaling activation [27]. Our results showed that TTM promoted stabilization of exogenous NRF2-Myc, whereas KEAP1-Flag overexpression accelerated degradation of NRF2-Myc in TTM-treated cells. More importantly, Keap1 C151S mutant further promoted NRF2-Myc degradation, while KEAP1 C151A mutant remarkably induced the accumulation of NRF2-Myc in TTM-treated cells (Fig. 2E). These results indicate that KEAP1 is implicated in NRF2 activation induced by TTM and KEAP1 cysteine 151 is a major sensor for TTM-mediated activation of NRF2 antioxidant signaling.

### TTM enhances autophagy flux in vascular endothelial cells

**Fig. 3:**
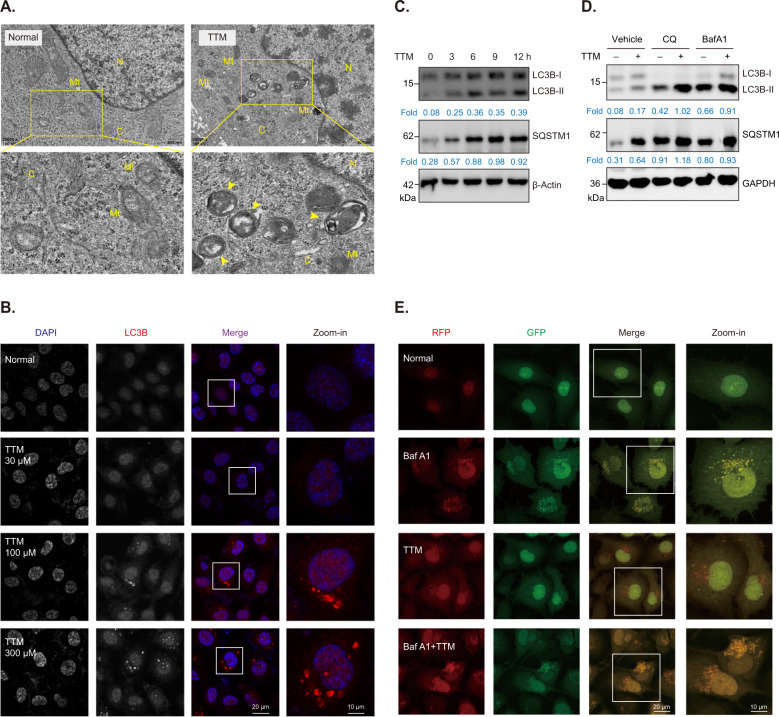
TTM activates autophagy signaling pathway in HUVECs. **A** Representative TEM images of TTM-treated HUVECs. Cells were treated with 100 μM TTM for 12 h. N, nucleus; C, cytoplasm; Mt, mitochondria; Yellow arrow, autophagosomes. **B** Representative immunofluorescence images of LC3B puncta in HUVECs treated with 30, 100, or 300 μM TTM for 12 h. Scale bar, 20 μm; Zoom-in scale bar, 10 μm. **C** Western blot analysis and quantification of LC3B and SQSTM1 in HUVECs treated with 100 μM TTM for 0, 3, 6, 9, and 12 h, respectively. β-Actin was used as loading control. **D** Western blot analysis and quantification of LC3B and SQSTM1 in TTM-treated HUVECs with or without lysosome inhibitor CQ (5 μM) and BafA1 (100 nM), respectively. GAPDH was used as loading control. **E** Autophagic flux assay using tandem fluorescent-tagged LC3B (RFP-GFP-LC3B) construct. Stable cell line expressing RFP-GFP-LC3B was pretreated with or without Baf A1 for 1 h and then co-incubated with TTM for 12 h. Scale bar, 20 μm; Zoom-in scale bar, 10 μm.

Autophagy is a highly conserved process that regulates degradation of misfolded protein and damaged organelles in a lysosome-dependent manner [28]. Taguchi et al. previously reported that autophagy regulates NRF2 signaling via promoting KEAP1 autophagic degradation [29]. Therefore, we explored whether autophagy modulates NRF2 activation induced by TTM. TEM images showed that TTM treatment significantly induced accumulation of autophagosomes (double membrane vesicles) in HUVECs (Fig. 3A). We further showed that TTM treatment increased LC3B (a reliable marker to labeling autophagosomes) fluorescence signal in HUVECs (Fig. 3B). In addition, we showed that TTM treatment obviously upregulated protein levels of LC3B-II in HUVECs and in mice liver tissues (Figs. 3C and S1B). Since the accumulation of LC3B-II and autophagosomes results mainly from either autophagy activation or impaired autophagy flux, we determined the autophagic flux in TTM-treated cells using lysosome inhibitors including chloroquine (CQ) and bafilomycin A1 (BafA1). Our results revealed that TTM increased protein levels of LC3B-II and SQSTM1, and these two proteins were further increased in cells co-treated with lysosome inhibitor and TTM, suggesting that TTM does enhance autophagic flux in HUVECs (Fig. 3D). Furthermore, we used a tandem RFP-GFP-LC3 construct to monitor autophagic flux. The GFP fluorescence is quenched at lysosome acidic conditions (pH < 5.5), whereas RFP fluorescence is not affected in acidic lysosomes. Accordingly, RFP-GFP-LC3 puncta shows both GFP and RFP fluorescence (merged as yellow LC3 dots) in autophagosomes, but exhibits only RFP fluorescence (RFP LC3 dots) in autolysosomes. Our results showed that TTM treatment remarkably increased RFP LC3 dots in HUVECs. Moreover, combined treatment with BafA1 and TTM increased more yellow LC3 dots than BafA1 separate treatment group, indicating that TTM stimulates autophagy but does not impairs autophagic flux in HUVECs (Fig. 3E). To conclude, these data demonstrate that TTM enhances autophagic flux in vascular endothelial cells.

### Autophagy modulates NRF2 activation induced by TTM

**Fig. 4:**
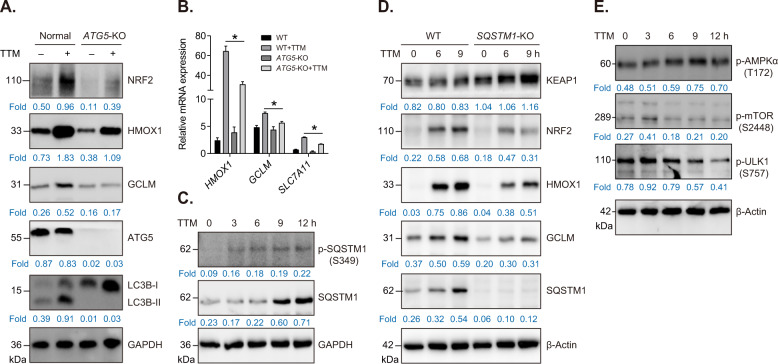
Autophagy contributes to NRF2 pathway activation in TTM-treated HUVECs. **A** Western blot analysis and quantification of NRF2, HMOX1, GCLM, ATG5, and LC3B in WT or *ATG5*-KO cells treated with or without 100 μM TTM for 12 h. GAPDH was used as loading control. **B** qPCR analysis for *HMOX1*, *GCLM*, and *SLC7A11* in WT and *ATG5*-KO cells after treatment with or without TTM. One-way ANOVA followed by a Tukey multiple comparison test was used for statistical analysis. *p < 0.05. **C** Western blot analysis and quantification of p-SQSTM1 (S349) and SQSTM1 in HUVECs treated with 100 μM TTM for indicated time. β-Actin was used as loading control. **D** Western blot analysis and quantification of KEAP1, NRF2, HMOX1, GCLM, and SQSTM1 in WT and *SQSTM1*-KO cells treated with TTM for indicated time. β-Actin was used as loading control. **E** Western blot analysis and quantification of p-AMPKα (T172), p-MTOR (S2488), and p-ULK1 (S757) in TTM-treated HUVECs. β-Actin was used as loading control.

To verify whether autophagy participates in TTM-induced NRF2 activation in HUVECs, we constructed a stable *ATG5* knockout (*ATG5*-KO) cell line using CRISPR/Cas9. We firstly verified the *ATG5* knockout efficiency and autophagic flux blockage in HUVECs (Fig. 4A). Then, the results revealed that the upregulation of NRF2 and its downstream molecules induced by TTM was significantly compromised in *ATG5*-KO cells (Fig. 4A). Subsequently, qRT-PCR results showed that *ATG5* knockout considerably repressed transcription of NRF2 target genes *HMOX1*, *GCLM*, and *SLC7A11* (Fig. 4B). Accumulating evidences have revealed that autophagy adapter SQSTM1/p62 is implicated in autophagy-medicated NRF2 activation. The phosphorylated SQSTM1 (at serine 349) shows high affinity to KEAP1, resulting in the release of NRF2 from KEAP1-NRF2 complex and activation of NRF2 antioxidant signaling pathway [30, 31]. In this study, we showed that TTM induced the phosphorylation of serine 349 of SQSTM1 in HUVECs (Fig. 4C). Furthermore, *SQSTM1* knockout resulted in significant accumulation of KEAP1 and prevented the activation of TTM-mediated NRF2 signaling pathway, suggesting that TTM activates NRF2 antioxidant pathway via SQSTM1-dependent manner (Fig. 4D). We further investigated the upstream signaling pathway that participates in TTM-regulated autophagy pathway. Immunoblotting results revealed that TTM increased phosphorylation level of p-AMPKα (T172), while decreased protein levels of p-mTOR (S2448) and p-ULK1 (S757) in HUVECs, indicating TTM induces autophagy through activating AMPK/mTOR/ULK1 pathway (Fig. 4E).

### TTM protects against NaAsO_2_-induced oxidative stress in an NRF2-dependent manner

**Fig. 5:**
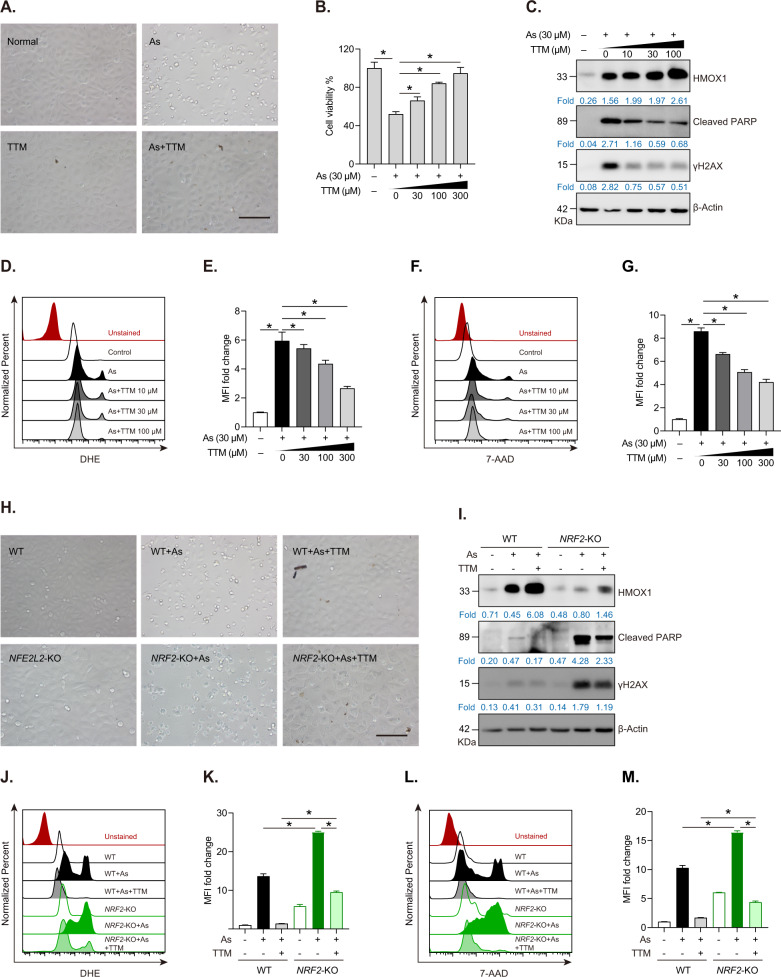
TTM-mediated NRF2 activation protects HUVECs from NaAsO_2_-induced oxidative damage. **A** Representative morphological changes of TTM-treated HUVECs with or without NaAsO_2_ (As, 30 μM) for 24 h. Scale bar, 100 μm. TTM and NaAsO2 were dissolved in culture media and simultaneously added to the cell culture plates. **B** Cell viability assay of HUVECs treated with NaAsO_2_ (30 μM) with or without different dose of TTM for 24 h. **C** Western blot analysis and quantification of HMOX1, cleaved PARP, γH2AX, and β-Actin in HUVECs treated with NaAsO_2_ (30 μM) and different dose of TTM for 24 h. **D**, **E** FACS analysis and quantification of DHE intensity in HUVECs treated with 30 μM NaAsO_2_ and different dose of TTM for 24 h. **F**, **G** FACS analysis and quantification of 7-AAD intensity in HUVECs treated with 30 μM NaAsO_2_ and different dose of TTM for 24 h. **H** Representative morphological changes of NaAsO_2_-treated WT or *NRF2*-KO with or without 100 μM TTM for 24 h. Scale bar, 100 μm. **I** Western blot analysis and quantification of HMOX1, cleaved PARP, γH2AX and β-Actin in WT and *NRF2*-KO cells treated with NaAsO_2_ and TTM plus NaAsO_2_. **J**, **K** FACS analysis and quantification of DHE intensity in WT and *NRF2*-KO cells treated with NaAsO_2_ and TTM plus NaAsO_2_. **L**, **M** FACS analysis and quantification of 7-AAD intensity in WT and *NRF2*-KO cells treated with NaAsO_2_ and TTM plus NaAsO_2_. MFI mean fluorescence intensity. One-way ANOVA followed by a Tukey multiple comparison test was used for statistical analysis. *p < 0.05.

Furthermore, we demonstrated the functional roles of TTM-regulated NRF2 signaling under oxidative injury induced by NaAsO_2_, a well characterized oxidative stress inducer. Cell morphology analysis and cell viability assay showed that NaAsO_2_ exposure obviously induced cell death in HUVECs characterized by cell shrinking, rounding up and falling off (Figs. 5A and S1D). Moreover, we showed that TTM protected HUVECs from NaAsO_2_-induced cellular damage (Fig. 5A). Our results further revealed that TTM considerably alleviated NaAsO_2_-induced apoptotic cell death in a dose-dependent manner (Figs. 5B and S1E). The results also showed that TTM treatment decreased protein levels of cleaved PARP (an apoptotic marker) and γH2AX (a sensor of DNA damage) induced by NaAsO_2_ exposure (Fig. 5C). FACS data showed that TTM decreased ROS levels and significantly repressed 7-AAD fluorescence (an apoptotic probe) in NaAsO_2_-treated HUVECs (Fig. 5D–G). More importantly, we demonstrated that *NRF2* knockout undermined protective effects of TTM against NaAsO_2_-induced cell death (Figs. 5H and S1F). Immunoblotting results revealed that *NRF2* knockout significantly elevated protein levels of cleaved PARP and γH2AX in cells co-treated with NaAsO_2_ and TTM (Fig. 5I). Consistently, FACS data demonstrated that *NRF2* knockout enhanced cellular oxidative stress and exacerbated cell death in cells co-treated with NaAsO_2_ and TTM (Fig. 5J–M). These data indicate that TTM alleviates NaAsO_2_-induced oxidative stress and cellular damage in an NRF2-dependent manner.

### Autophagy-dependent NRF2 activation contributes to the protective role of TTM against NaAsO_2_-induced oxidative injury

**Fig. 6:**
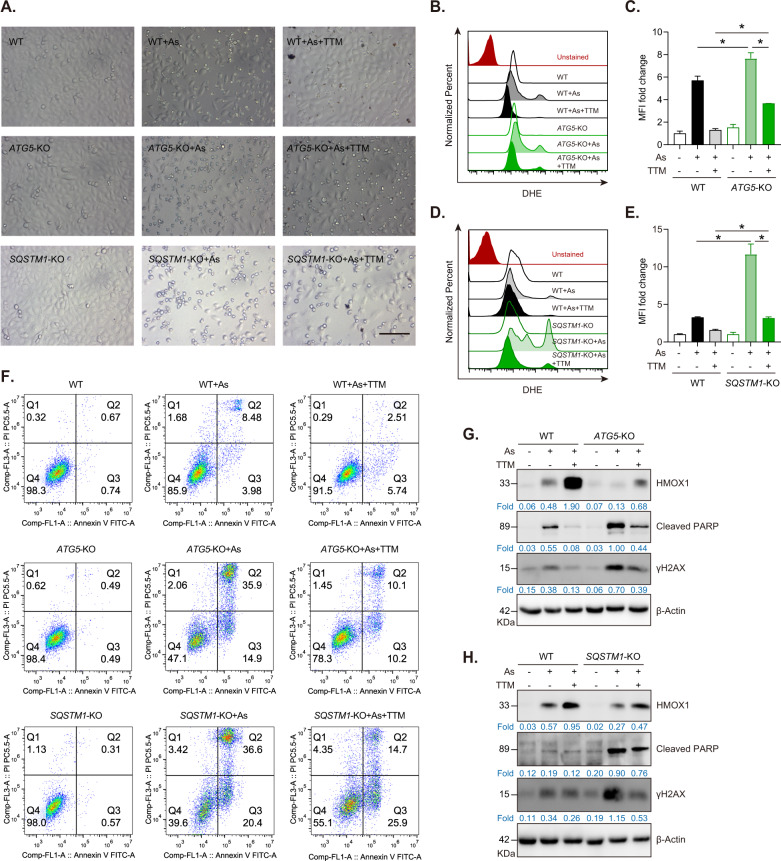
Autophagy-dependent activation of NRF2 protects HUVECs from NaAsO_2_-induced cytotoxicity. **A** Representative morphological changes of WT control, *ATG5*-KO or *SQSTM1*-KO cells treated with 30 μM NaAsO_2_ with or without 100 μM TTM for 24 h, respectively. Scale bar, 100 μm. **B**, **C** FACS analysis and quantification of DHE intensity in WT control or *ATG5*-KO cells treated with 30 μM NaAsO_2_ with or without 100 μM TTM for 12 h. **D**, **E** FACS analysis and quantification of DHE intensity in WT control or *SQSTM1*-KO cells treated with 30 μM NaAsO_2_ with or without 100 μM TTM for 12 h. In **C** and **E**, MFI mean fluorescence intensity. One-way ANOVA followed by a Tukey multiple comparison test was used for statistical analysis. *p < 0.05. **F** FACS analysis of apoptotic cell death in WT control, *ATG5*-KO and *SQSTM1*-KO cells treated with 30 μM NaAsO_2_ with or without 100 μM TTM for 24 h. **G** Western blot analysis and quantification of HMOX1, cleaved PARP, γH2AX, and β-Actin in WT and *ATG5*-KO cells treated with NaAsO_2_ with or without TTM. **H** Western blot analysis and quantification of HMOX1, cleaved PARP, γH2AX and β-Actin in WT control or *SQSTM1*-KO cells treated with NaAsO_2_ with or without TTM.

To investigate whether autophagy contributes to TTM antioxidant effects, we investigated the protective effects of TTM on NaAsO_2_-induced oxidative stress in *ATG5* knockout and *SQSTM1* knockout cells. Results showed that autophagy impairment by knocking out *ATG5* or *SQSTM1* weakened the protective effects of TTM and exacerbated NaAsO_2_-induced cell death in TTM-treated HUVECs (Fig. 6A). FACS data confirmed that *ATG5* knockout or *SQSTM1* knockout increased oxidative stress, while TTM can significantly mitigate NaAsO_2_-induced oxidative injury in wild type cells but not in *ATG5*-KO or *SQSTM1*-KO cells (Fig. 6B–E). Our results also showed that the blockade of autophagy exacerbated NaAsO_2_-induced apoptotic cell death (Fig. 6F). Immunoblotting results further verified that *ATG5* or *SQSTM1* knockout undermined the protective role of TTM against NaAsO_2_-induced DNA damage and apoptotic cell death in HUVECs (Fig. 6G, H). Taken together, our data demonstrate that autophagy-dependent NRF2 activation contributes to the protective role of TTM against NaAsO_2_-induced oxidative injury.

## Discussion

Ammonium tetrathiomolybdate (TTM) is a clinical copper chelator and an inhibitor of copper trafficking proteins that has potential in treating copper storage diseases [32]. In the current study, we identified an unknown characteristic of TTM that served as a novel NRF2 activator, which was distinctive from previous studies that recognizing TTM only as a copper chelator. We demonstrated that TTM promoted NRF2 nuclear translocation in vascular endothelial cells and obviously upregulated the transcriptional level of NRF2 downstream antioxidative genes, including *HMOX1*, *GCLM*, and *SLC7A11*. (Fig. 1). Importantly, we firstly demonstrated that TTM exhibited protective effects on sodium arsenite (NaAsO_2_)-induced oxidative stress (Fig. 5A–G). Furthermore, we confirmed that TTM-mediated NRF2 signaling activation contributed to the protective effects of TTM in that *NRF2* knockout exacerbated NaAsO_2_-induced oxidative damage and apoptotic cell death in HUVECs (Fig. 5H–M). TTM is a sulfur-containing compound and shares similar chemistry properties to other NRF2 activator such as sulforaphane and oltipraz [33]. Previous studies have demonstrated that sulforaphane and oltipraz are hydrogen sulfide (H_2_S) donors, which exhibits promising protective effects through release of H_2_S [34]. Intriguingly, Alex Dyson et al. have also revealed that TTM is a new class of hydrogen sulfide donor [35, 36]. Hydrogen sulfide increases the nuclear localization of NRF2 and functions as a cardioprotective signaling molecule [37, 38]. In parallel, other studies have demonstrated that H_2_S induces the S-sulfhydration of KEAP1 at Cys151, contributes causally to NRF activation and alleviates multiple diseases, such as diabetic atherosclerosis, liver injury and cellular senescence [39–41]. Taken together, we speculate that TTM induces NRF2 activation probably through releasing H_2_S in HUVECs.

Autophagy is known as a conserved catabolic process involved in removing unnecessary or macromolecules and organelles [42]. Herein, we demonstrated that copper chelator TTM activated autophagy pathway in HUVECs (Fig. 3). It has been reported that triethylenetetramine (trientine), another FDA-approved copper-chelating agent, enhances autophagic flux in hepatocytes via stabilizing spermidine acetyltransferase 1 and reducing cellular protein acetylation [43]. These findings are consistent with a study which demonstrates that TTM treatment or the knockdown of copper transporter SLC31A1 (also called Ctr1) significantly increases the ratio of LC3B-II/I and promoted autophagy in pancreatic cancer cells [44]. In addition, TTM-derived H_2_S was also reported as an autophagy activator, which initiated autophagy via S-sulfhydration modification of autophagy master regulator TFEB or autophagy regulator GAPDH [45, 46]. Subsequently, we investigated the signal pathways that regulated autophagy in TTM-treated HUVECs. We showed that TTM induced the phosphorylation level of p-AMPKα (T172), but significantly decreased the protein levels of p-mTOR (S2448) and p-ULK1 (S757) in HUVECs (Fig. 4E). Consistently, a research group revealed that TTM-mediated copper deficiency triggered AMPK while suppressed mTORC1 signaling in breast cancer cell line MDA231-LM2 [47]. Similar results were also observed on activating AMPK signaling with TTM in neuroblastoma cell line SH-SY5Y [48]. These results suggest that TTM activates autophagy through AMPK/mTOR/ULK1-dependent pathway.

Followingly, we revealed the implications of autophagy in TTM-mediated NRF2 signaling activation. Previous studies have revealed that the autophagy adapter SQSTM1 links autophagy and NRF2 signaling [30]. Phosphorylated SQSTM1 at serine 349 (serine 351 in mouse) has higher affinity for KEAP1 and can competitively bind with KEAP1, resulting in NRF2 signaling activation as the release of NRF2 from KEAP1-NRF2 protein complex [31]. Moreover, autophagy deficiency activates the NRF2 pathway since the excessive accumulation of SQSTM1 due to autophagy inhibition competes with NRF2 for binding to KEAP1, thus resulting in activation of NRF2 signaling pathway [49]. Unexpectedly, in this study, our results revealed that autophagy deficiency induced by knockout of *ATG5* remarkably decreased NRF2 level and tremendously inhibited the transcription level of NRF2 target genes in TTM-treated HUVECs, including *HMOX1*, *GCLM*, and *SLC7A11*. This finding indicates autophagy does not block but promote NRF2 signaling activation in TTM-treated HUVECs. In agreement with our results, several previous studies have also demonstrated that autophagy positively correlates with activation of NRF2 signaling and transcription upregulation of downstream antioxidant genes [50–52]. Mechanistically, we revealed that TTM treatment induced the phosphorylation of serine 349 of SQSTM1 (Fig. 4C) and promoted autophagic degradation of KEAP1 in HUVECs (Fig. 4D). Moreover, we and others have demonstrated that autophagy deficiency reciprocally activates ubiquitin-proteasome pathway and accelerates proteasomal degradation of NRF2 [24, 53]. Therefore, we speculate that autophagy and SQSTM1 contribute to TTM-mediated NRF2 activation via simultaneously inducing the release of NRF2 from the KEAP1-NRF2 complex and preventing NRF2 degradation in proteasome in HUVECs.

Inorganic arsenic (NaAsO_2_) is a class of environmental pollutants mainly derived from coal burning, non-ferrous metals production, and polluted water [54]. It has been demonstrated that arsenic exposure is significantly associated with an increased risk of cardiovascular disease and coronary heart disease [55]. Arsenic is also used as oxidative stress inducer to induce excessive ROS production and cellular oxidative DNA damage [56]. In this work, we demonstrated a new use for the old drug TTM in alleviating NaAsO_2_ exposure-induced vascular toxicity. Our results showed that TTM considerably alleviated NaAsO_2_-induced oxidative stress and cellular damage in HUVECs (Fig. 5A–G). Importantly, we revealed that *NRF2* knockout partially weakened TTM protective effects against NaAsO_2_-induced cytotoxicity in HUVECs, indicating that TTM-initiated NRF2 signaling pathway participated in mitigating arsenic-induced cardiovascular toxicity. However, it should also be noted that TTM still partially alleviate NaAsO_2_-induced oxidative stress and cell death in the NRF2, ATG5, or SQSTM1-deficiency cells, suggesting that there are other molecular mechanisms that have contributed to the protective effects of TTM against NaAsO_2_ induced toxicity (Figs. 5–6). In clinical, metal chelators, such as sodium 2,3-dimercapto-1-propanesulfonate (Unithiol), D-penicillamine and dimercaptosuccinic acid (Succimer), are applied as effective strategy against arsenic toxicity, suggesting that TTM may also play protective role in vascular endothelial cells via chelating excessive toxic arsenic [57, 58]. In addition, we and others have uncovered that arsenic exposure causes mitochondrial oxidative damage and ferroptosis characterized by iron overload-induced lipid peroxidation [59, 60]. As hydrogen sulfide can attenuate ferroptotic cell death [61, 62], we speculate that the hydrogen sulfide donor TTM can also inhibit NaAsO_2_-induced ferroptosis. Recently, Tsvetkov et al. founded a novel copper-dependent death named cuproptosis and showed that copper chelator TTM rescued cells from copper overload-induced cell death [63]. It is still an open question whether TTM protects against arsenic-induced vascular injury via reducing cuproptosis in vascular endothelial cells. We expect to elucidate this issue in our ongoing work.

## Conclusion

In this work, we demonstrated that copper chelator TTM is a novel antioxidant regent, which induced NRF2 nuclear translocation and activated downstream antioxidative genes transcription. Mechanistically, TTM activated NRF2 signaling via AMPK/mTOR/ULK1-dependent autophagy pathway. Moreover, TTM-mediates NRF2 signaling protects against arsenic-induced cytotoxicity, indicating that TTM is a promising therapeutic agent against oxidative stress-induced vascular diseases.

## Supplementary information

Supplemental Material

Original Data File

Reproducibility checklist

## Data Availability

The supporting data are available from the corresponding authors on request.
