# Supplementary material for: Ammonium tetrathiomolybdate triggers autophagy-dependent NRF2 activation in vascular endothelial cells
Source: Cell Death Dis. 2022 Aug 25;13(8):733. doi: 10.1038/s41419-022-05183-z (PMC9411162; doi:10.1038/s41419-022-05183-z)
Supplement: Supplementary file 1 — Supplemental Material [file 41419_2022_5183_MOESM1_ESM.pdf]

Supplementary Figure Captions

Figure S1.

(A) Cell viability assay of HUVECs treated with TTM (10, 30, 100, 300 or 1,000 μM) for 24 h. **(B)** Female C57BL/6J mice were gavaged with 30 mg/kg of TTM twice a day for seven consecutive days. Western blot analysis and quantification of NRF2, LC3B and β-Actin in in liver tissues. **(C)** Liver tissues from TTM-treated mice were subjected to immunohistochemistry analysis. Representative images of immunohistochemistry of paraffin-embedded mice liver tissues using antibodies against NRF2. Antigen retrieval was perfomed using Tris-EDTA buffer (pH 9). **(D)** Cell viability assay of HUVECs treated with $NaAsO_2$ (10, 20, 30, 100, 200 or 300 μM) for 24 h. **(E)** FACS analysis of apoptotic cell death in HUVECs treated with 30 μM $NaAsO_2$ with or without TTM for 24 h. **(F)** FACS analysis of apoptotic cell death in WT control and *NRF2*-KO cells treated with 30 μM $NaAsO_2$ with or without 100 μM TTM for 24 h. In **(A)** and **(D)**, Student's *t*-test was used for statistical significance. $^*p<0.05$ was considered statistical significance.

Figure S1.

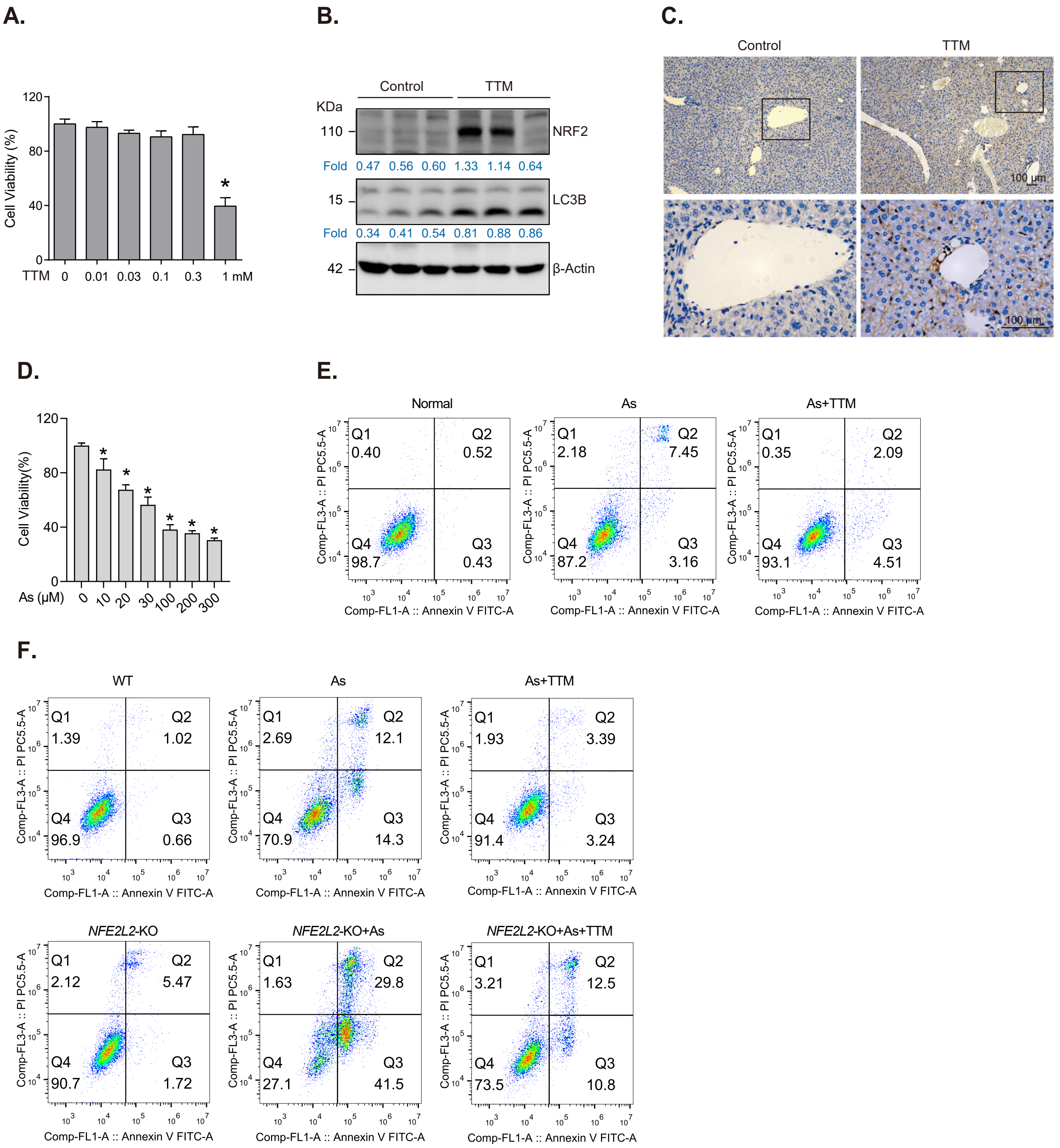
A.
Cell Viability (%)
TTM 0 0.01 0.03 0.1 0.3 1 mM
B.
Control TTM
KDa
110 NRF2
Fold 0.47 0.56 0.60 1.33 1.14 0.64
15 LC3B
Fold 0.34 0.41 0.54 0.81 0.88 0.86
42 β-Actin
C.
Control TTM
100 μm
D.
Cell Viability(%)
As (μM) 0 10 20 30 100 200 300
E.
Normal As As+TTM
Q1 0.40 Q2 0.52 Q4 98.7 Q3 0.43
Q1 2.18 Q2 7.45 Q4 87.2 Q3 3.16
Q1 0.35 Q2 2.09 Q4 93.1 Q3 4.51
Comp-FL3-A :: PI PC5.5-A
Comp-FL1-A :: Annexin V FITC-A
F.
WT As As+TTM
Q1 1.39 Q2 1.02 Q4 96.9 Q3 0.66
Q1 2.69 Q2 12.1 Q4 70.9 Q3 14.3
Q1 1.93 Q2 3.39 Q4 91.4 Q3 3.24
NFE2L2-KO NFE2L2-KO+As NFE2L2-KO+As+TTM
Q1 2.12 Q2 5.47 Q4 90.7 Q3 1.72
Q1 1.63 Q2 29.8 Q4 27.1 Q3 41.5
Q1 3.21 Q2 12.5 Q4 73.5 Q3 10.8
Comp-FL3-A :: PI PC5.5-A
Comp-FL1-A :: Annexin V FITC-A
