# Supplementary material for: Ammonium tetrathiomolybdate triggers autophagy-dependent NRF2 activation in vascular endothelial cells
Source: Cell Death Dis. 2022 Aug 25;13(8):733. doi: 10.1038/s41419-022-05183-z (PMC9411162; doi:10.1038/s41419-022-05183-z)

Original Data File

Figure 1.

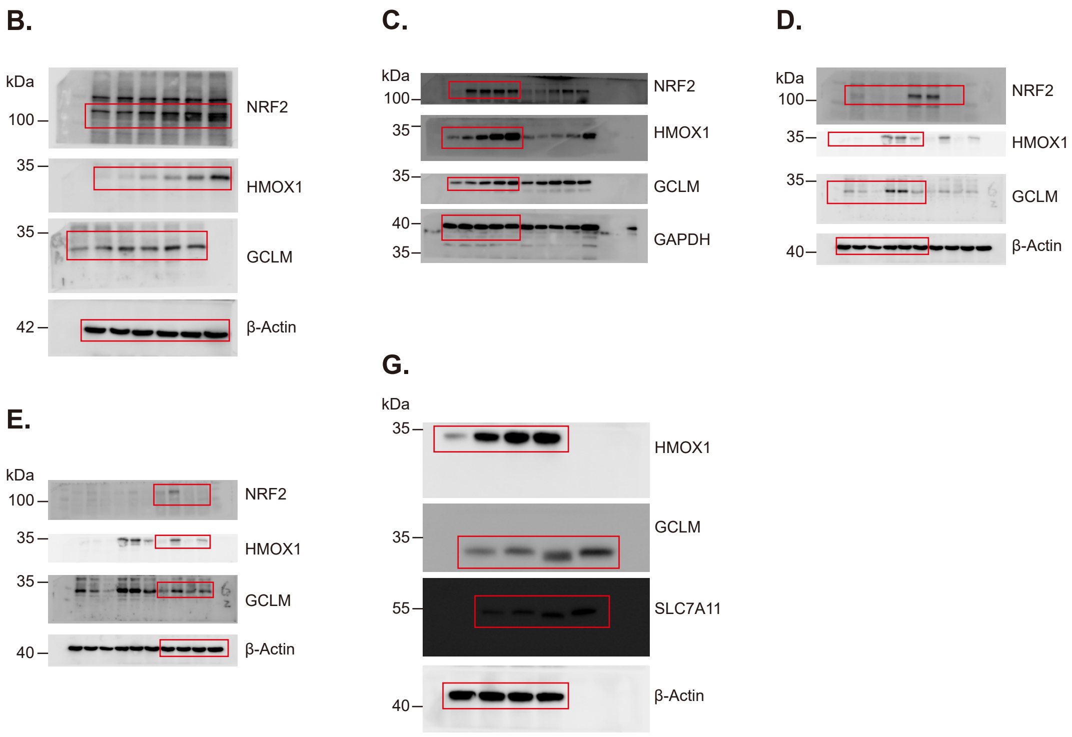

Figure 2.

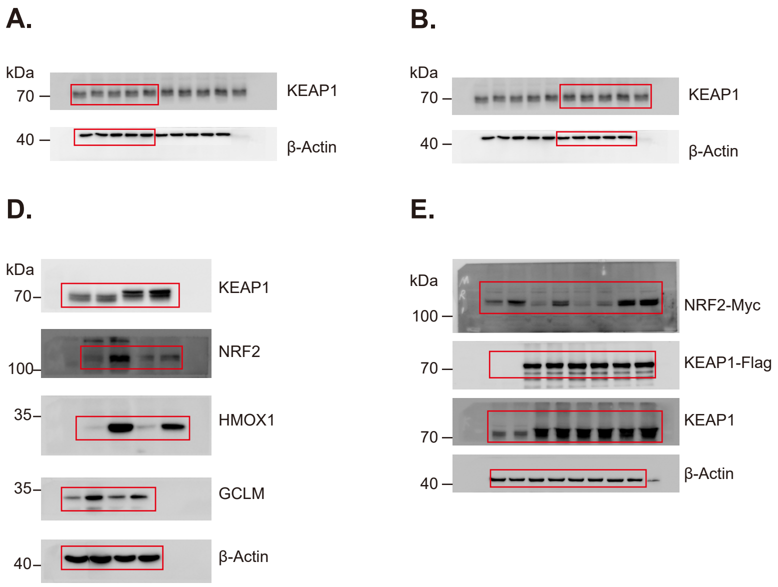

Original Data File

Figure 3.

C.

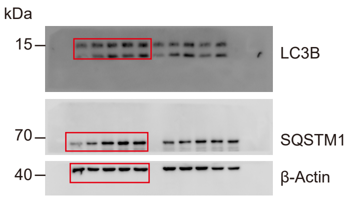

D.

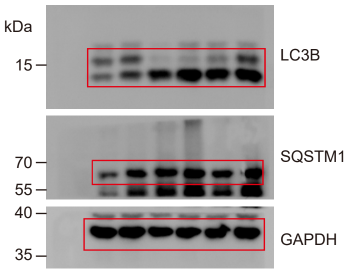

Figure 4.

A.

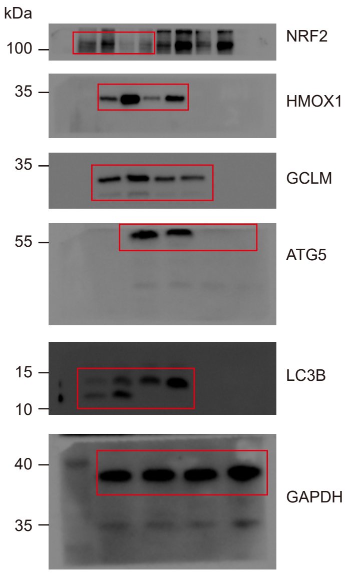

C.

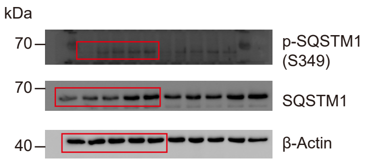

D.

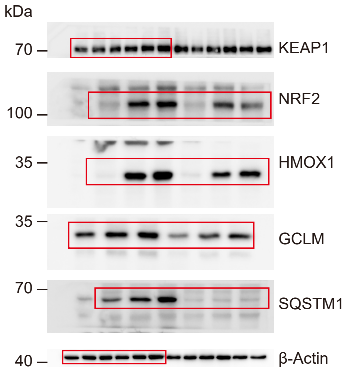

E.

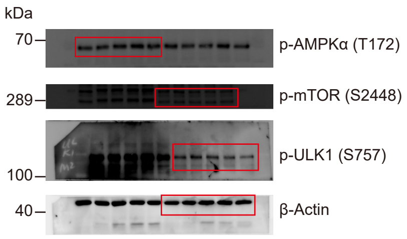

Original Data File

Figure 5.

C.

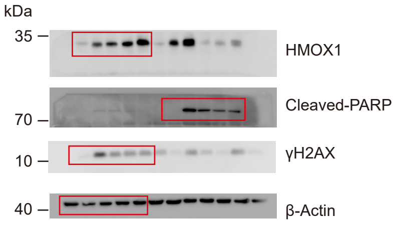

I.

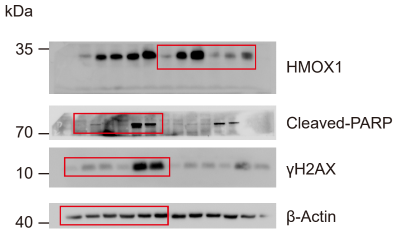

Figure 6.

G.

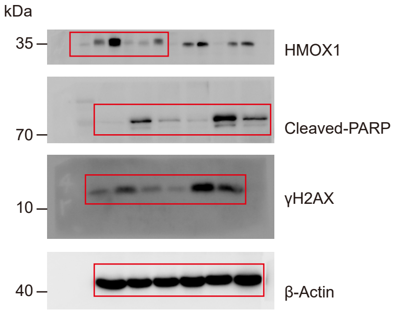

H.

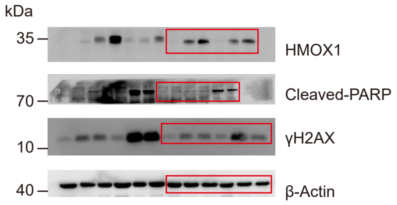

Figure S1.

B.

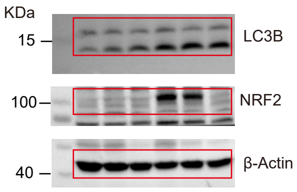

Supplement: Supplementary file 2 — Original Data File [file 41419_2022_5183_MOESM2_ESM.pdf]
